# Early clinical and radiographic outcomes of robot-assisted femoral neck system fixation combined with an additional cannulated compression screw for garden IV femoral neck fractures in young and middle-aged adults: a propensity score-matched retrospective cohort study

**DOI:** 10.3389/fsurg.2026.1958968

**Published:** 2026-10-19

**Authors:** Wu Fengqin, Aihelamu Tueraili, Yaqiong Zhao

**Affiliations:** The Sixth Affiliated Hospital of Xinjiang Medical University, Urumqi, China

**Keywords:** cannulated compression screw, Femoral neck fracture, Femoral neck shortening, Femoral Neck System, Hip preservation, Propensity score matching, Robot-assisted surgery

## Abstract

**Background:**

Garden IV femoral neck fractures in young and middle-aged adults are highly unstable and prone to shortening, nonunion, and femoral head necrosis. We compared robot-assisted femoral neck system (FNS) fixation plus an additional cannulated compression screw (CCS) in a multiplanar configuration with robot-assisted FNS fixation alone.

**Methods:**

This retrospective cohort included 172 patients aged 18-60 years who were treated from June 2024 to June 2025; 82 underwent robot-assisted FNS plus CCS fixation and 90 underwent robot-assisted FNS fixation alone. After 1:1 nearest-neighbor propensity score matching without replacement (caliper, 0.2 standard deviations of the logit propensity score), 67 matched pairs were analyzed. Femoral neck shortening at 12 months was defined as the primary analytic endpoint for this report; perioperative, rehabilitation, other radiographic, functional, and complication outcomes were secondary or exploratory.

**Results:**

FNS plus CCS required longer operative time (81.67 ± 7.62 vs. 68.67 ± 7.51 min), more fluoroscopic exposures and guidewire adjustments, and a longer total incision (all P < 0.05). It was associated with earlier full weight-bearing (10.74 ± 1.42 vs. 12.47 ± 1.34 weeks), shorter fracture-healing time (4.69 ± 0.52 vs. 5.25 ± 0.43 months), less femoral neck shortening at 12 months (4.46 ± 1.35 vs. 6.08 ± 1.31 mm), and a lower rate of moderate shortening (34.3% vs. 73.1%; all P < 0.001). HHS was higher at 6 and 12 months and VAS was lower at 12 months in the combined group. Any complication occurred in 1.5% versus 10.4% (exact *P* = 0.070; conditional matched OR, 0.14; exact 95% CI, 0.003-1.11), indicating substantial uncertainty in the safety comparison.

**Conclusions:**

In this propensity score-matched retrospective cohort, robot-assisted FNS plus an additional CCS was associated with less femoral neck shortening, earlier full weight-bearing, shorter fracture-healing time, and better later functional recovery, at the cost of greater operative complexity. Because the safety analysis was underpowered and follow-up was limited to 12 months, these findings should be interpreted cautiously and do not establish long-term safety or femoral head necrosis risk; prospective multicenter studies with longer follow-up are required.

## Introduction

## Background

Garden IV fractures of the femoral neck in younger and middle-aged adults are typically due to high-energy trauma and are characterized by total displacement, significant shear forces, and extreme disruption of the femoral head blood supply (1).In a completely displaced fracture, the perfusion of the femoral head relies primarily on the terminal branches of the medial femoral circumflex artery; the displacement of the fracture may easily disrupt these vessels and raise the risk of avascular necrosis. Hip preservation is highly recommended in this population but existing internal fixation techniques cannot reliably strike a good balance between anatomy restoration, mechanical stability and maintenance of the remaining blood flow. Nonunion and necrosis of the femoral head are therefore important clinical problems (2).

The femoral neck system (FNS) is biomechanically superior to typical cannulated screws. It has a sliding bolt which is dynamically compressed and an antirotation screw that prevents the rotational movement. Biomechanical studies have demonstrated higher axial strength and torsional rigidity and better resistance to shear (1, 3).There are also meta-analyses indicating that FNS fixation can decrease operative time, complications, healing time, and femoral neck shortening and increase Harris Hip Scores compared to cannulated screws (4).However, a design involving such a simple bolt and antirotation screw might not offer adequate control of the multidirectional rotational instability and shortening of Garden IV fractures. The concept of three-dimensional stability would allow adding a CCS in another plane, which could add extra rotational and shear resistance and form a multiplanar stable construct (5).Such configuration has been found to minimize peak stress at the fracture location and restrict fragment displacement (6, 7).Similar reports of more rapid healing, reduced shortening and improved hip function have been noted with FNS plus CCS fixation but successful placement demands a high degree of accuracy of screw placement (8).Inserting several implants into the narrow corridor of the femoral neck is difficult to do accurately using a conventional freehand technique. Orthopaedic robotic navigation enables precise three-dimensional planning and placement of guidewires, eliminates repetitive drilling, which can further compromise the remaining blood flow, and is able to recreate the desired spatial design (9).

## Methods

### Study design and participants

The present retrospective cohort study included patients who had Garden IV femoral neck fractures that were managed in the Department of Orthopaedics of our facility during June 2024-June 2025. The inclusion criteria were: (1) patient age: 18-60 years; (2) radiological diagnosis of Garden IV femoral neck fracture; (3) management using robot-assisted FNS fixation or robot-assisted FNS and CCS fixation; and (4) presence of complete clinical and follow-up data. The exclusion criteria were: (1) pathological fracture or associated bone tumor; (2) neglected femoral neck fracture, defined as an injury-to-surgery interval of more than 3 weeks(3) severe osteoporosis (T-score less than −2.5); (4) severe craniocerebral trauma, polytrauma, or organ failure that rules out surgery; (5) prior ipsilateral hip surgery; and (6) follow-up less than 12 months.

Patients were divided into groups based on the fixation method, which included the FNS plus CCS group (the combined group) and the FNS-alone group. The initial cohort consisted of 172 patients (82 in the FNS plus CCS group and 90 in the FNS-alone group). The Ethics Committee of the Sixth Affiliated Hospital of Xinjiang Medical University approved the study (approval no. LFYLLSC20240111-12). Written informed consent for participation and use of clinical and imaging data was obtained from every participant; informed consent was not waived.

### Propensity score matching

PSM was used to reduce treatment-selection bias and confounding. The prespecified matching variables were age, sex, body mass index (BMI), mechanism of injury, injury-to-surgery interval, fracture side, Pauwels angle, Pauwels classification, and posterior femoral neck cortical comminution. One-to-one nearest-neighbor matching without replacement was performed, with exact stratification by mechanism of injury and a caliper width equal to 0.2 standard deviations of the logit of the propensity score. Sixty-seven matched pairs (134 patients) were retained for the final comparative analysis. Fifteen patients in the FNS plus CCS group and 23 in the FNS-alone group were excluded because no suitable match was identified (Figure 1).

**Figure 1:**
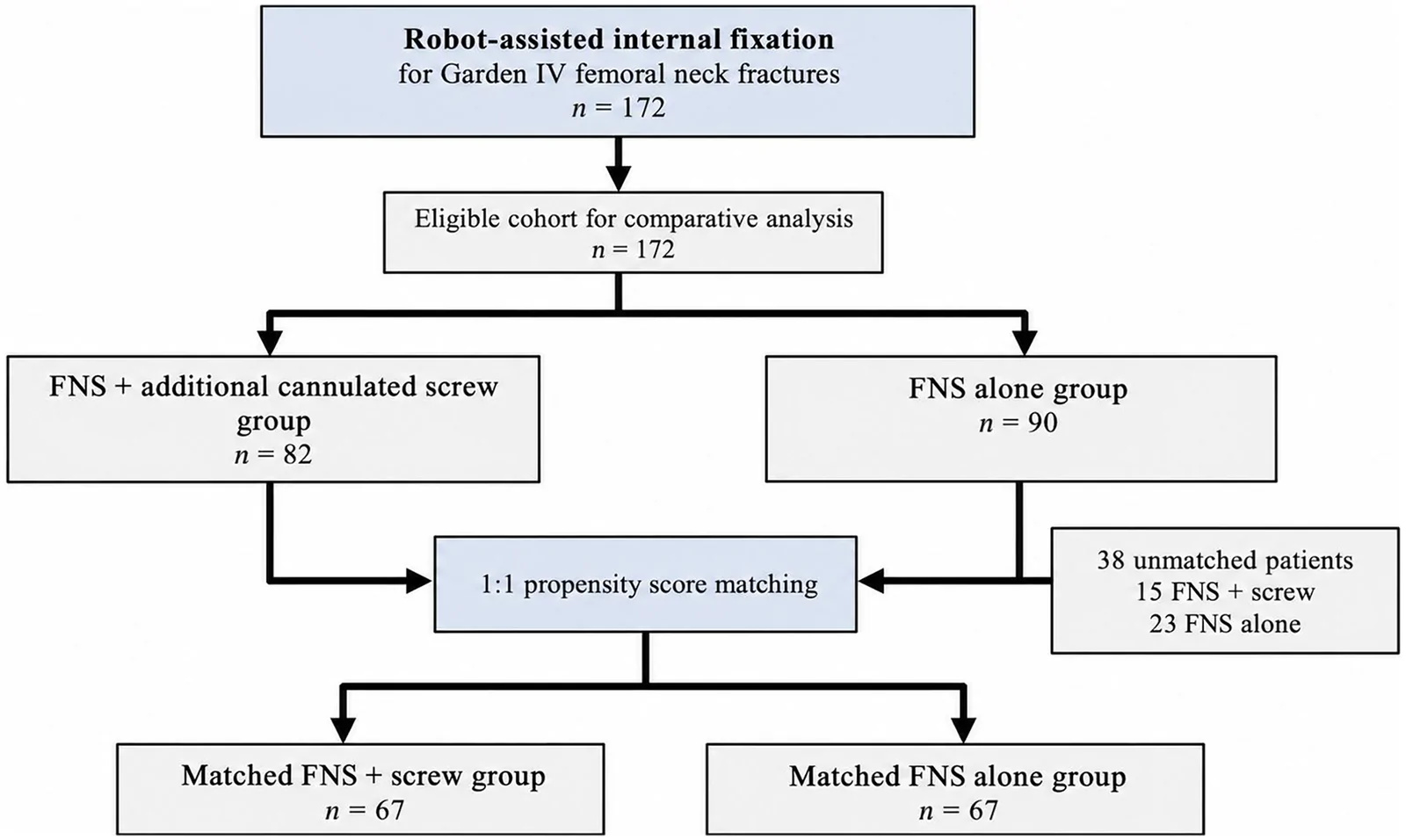
Patient selection and propensity score matching flow diagram. The initial cohort included 172 patients. After 1:1 propensity score matching, 67 matched pairs were included in the final analysis. FNS, femoral neck system; CCS, cannulated compression screw.

### Surgical procedures

The whole procedures were carried out by the same senior surgical team. The patients were placed in the supine position on a traction table. The reduction was done under C-arm fluoroscopy, and it aimed at obtaining anatomic or near-anatomic reduction, which is similar to Garden alignment index grade I or II. All operations involved an orthopaedic robot navigation system (TiRobot;Beijing Tianzhihang Medical Technology Co., Ltd.).

The preoperative three-dimensional computed tomography information on the affected proximal femur was loaded into the robotic surgical planning system. The entry point, insertion angle, implant length, and spatial positioning of the FNS bolt, antirotation screw, and additional CCS (combined group) were individualized according to the femoral neck axis and fracture morphology. For the additional CCS, the planned trajectory was required to lie in a plane distinct from the FNS bolt, usually anterior or superior, and to avoid implant intersection on anteroposterior and lateral/oblique projections. Because anatomy and fracture morphology varied, no single fixed insertion angle, screw length, or bolt-to-CCS distance was used across all patients. Following tracker placement and image registration, the robot arm guided each guidewire along the planned trajectory. The additional CCS was a 6.5-mm partially threaded cannulated compression screw (DePuy Synthes, Raynham, MA, USA) with a length ranging from 75 to 110 mm. The final screw length was selected intraoperatively according to the femoral neck anatomy and fracture morphology.

In the combined group, the FNS bolt was positioned centrally or slightly inferiorly in the femoral neck, and the antirotation screw was inserted through the FNS sleeve. One additional CCS was then inserted in another plane, usually anterior or superior to the FNS bolt. The bolt, antirotation screw, and CCS were arranged in a spatially crossed configuration to create multiplanar stability (Figure 2). In the FNS-alone group, only the FNS bolt and antirotation screw were implanted. Final fluoroscopy was used to confirm implant position and reduction, after which the wound was irrigated and closed in layers.

**Figure 2:**
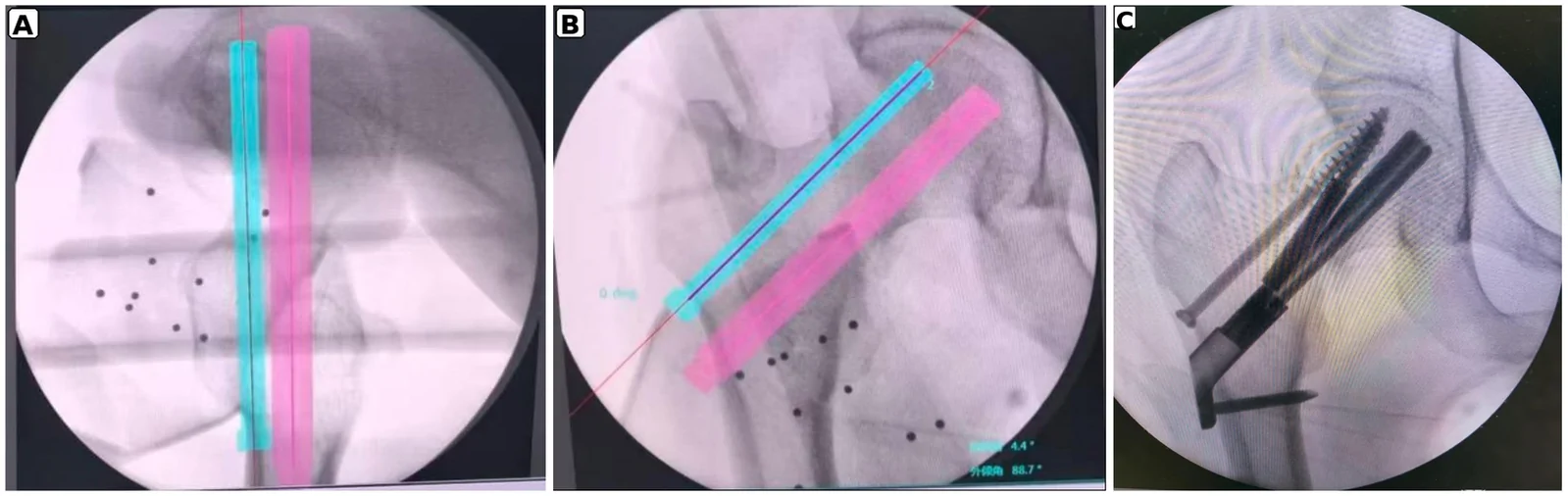
Robot-assisted placement of the multiplanar FNS-plus-CCS construct. **(A)** Anteroposterior fluoroscopic view showing the planned implant trajectories. **(B)** Oblique/lateral fluoroscopic view confirming spatial separation of the FNS and additional CCS. **(C)** Final fluoroscopic image showing the completed crossed multiplanar construct. FNS, femoral neck system; CCS, cannulated compression screw.

### Postoperative management and rehabilitation

Routine postoperative management included antibiotic prophylaxis, analgesia, and anticoagulation with low-molecular-weight heparin or rivaroxaban to prevent lower-extremity deep vein thrombosis. Quadriceps isometric exercises and ankle-pump exercises were initiated on postoperative day 1. The same staged rehabilitation framework was applied in both groups: depending on intraoperative stability and fracture pattern, patients began non-weight-bearing ambulation with two crutches at 2-4 weeks; weight-bearing was then advanced from partial to full only after serial radiographs demonstrated callus formation. The exact timing of progression remained individualized by the treating surgeon. Because the treating team knew the fixation method and the implant configuration was visible on radiographs, rehabilitation decisions were not blinded. Time to full weight-bearing was therefore regarded as a potentially clinician-influenced outcome.

### Outcome measures

Femoral neck shortening at 12 months was defined as the primary analytic endpoint for this report. Secondary or exploratory outcomes included operative time, intraoperative blood loss, number of fluoroscopic exposures, number of guidewire adjustments, total incision length, length of hospital stay, time to full weight-bearing, fracture-healing time, neck-shaft angle change, reduction quality, fracture union, moderate shortening, HHS, VAS, and postoperative complications.

Radiographic follow-up included anteroposterior pelvic and lateral hip radiographs at 1, 3, 6, and 12 months and at the final follow-up. Fracture union was defined by blurring or disappearance of the fracture line, continuous callus across the fracture site, and the absence of local tenderness or pain on axial percussion. Healing time was defined as the interval from surgery to the first follow-up at which these union criteria were met and was calculated only for fractures that united. Femoral neck shortening at 12 months and change in the neck-shaft angle were measured using the picture archiving and communication system and compared with immediate postoperative radiographs. Reduction quality was assessed using the Garden alignment index and categorized as excellent (160°-170° on the anteroposterior view and 160°-180° on the lateral view), good (150°-160° on either view), or poor (<150° or >180° on either view). Nonunion and moderate femoral neck shortening (5-10 mm) were recorded. Formal blinding of outcome assessors was not documented in the retrospective records; radiographic blinding was not feasible because the implant configuration was visible.

Femoral neck shortening was measured independently by two senior orthopedic surgeons at the associate-chief-physician level, both experienced in radiographic assessment, using previously reported methods (10–13). For the FNS bolt, X0 was the immediate postoperative distance from the bolt tip to the lateral plate and D0 was the radiographic bolt diameter; Xn and Dn were the corresponding measurements at each follow-up, and DR was the known actual bolt diameter. The calibrated sliding distance was calculated as XR = (X0/D0—Xn/Dn)  ×  DR (Figures 3A,B). For the CCS protrusion method, a was the radiographic screw-head thickness, b was the exposed screw length, and c was the known actual screw-head thickness; shortening was calculated as b  ×  c/a (Figure 3C). Both methods used known implant dimensions for calibration to reduce radiographic magnification error. When both measurements were available, the larger value was retained consistently as a conservative analytic rule to avoid underestimating clinically relevant shortening; this rule was not prospectively registered. Interobserver and intraobserver intraclass correlation coefficients were not available for this retrospective dataset. Residual error related to radiographic rotation and projection angle therefore cannot be excluded.

**Figure 3:**
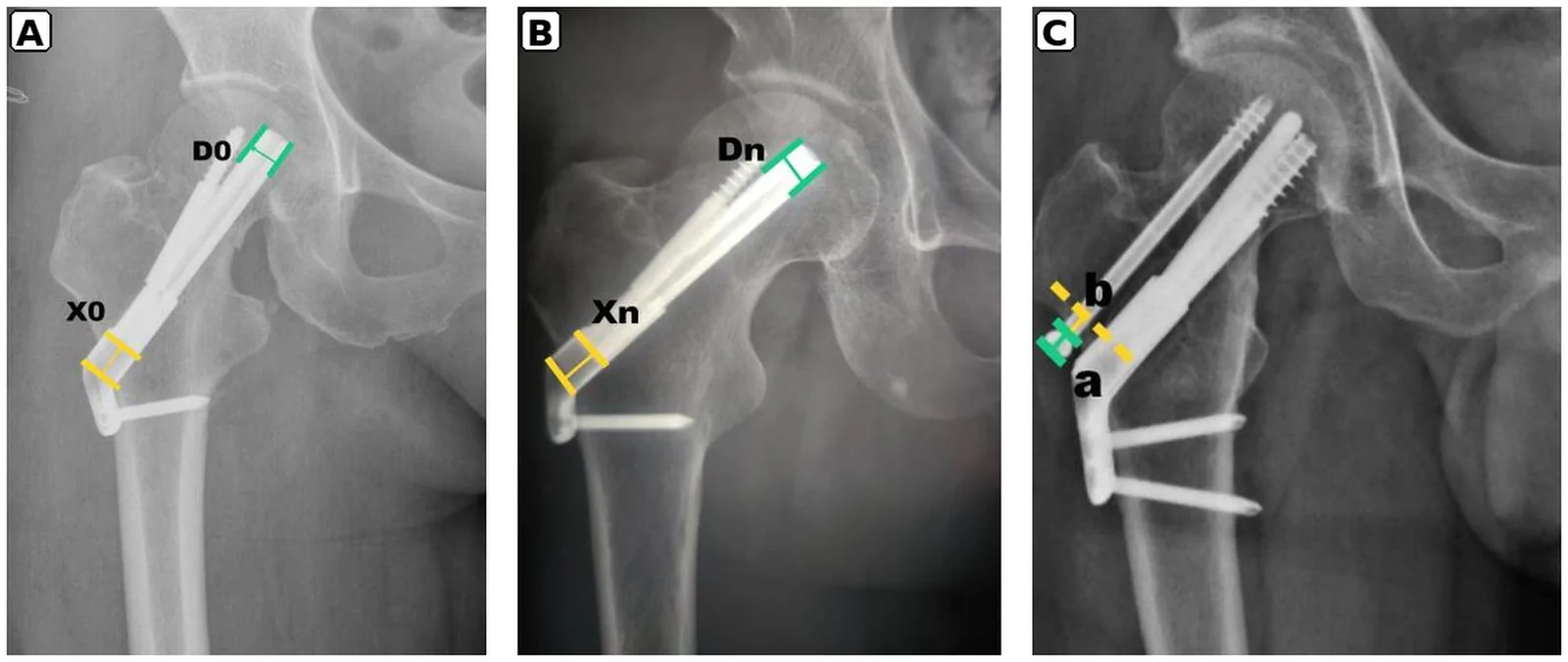
Radiographic measurement of femoral neck shortening. **(A)** Immediate postoperative measurement of the distance from the FNS bolt tip to the lateral plate (X0) and the apparent bolt diameter (D0). **(B)** Follow-up measurements (Xn and Dn). The calibrated sliding distance was XR = (X0/D0—Xn/Dn)  ×  DR, where DR is the actual bolt diameter. **(C)** Cannulated-screw protrusion method: a, radiographic screw-head thickness; b, exposed screw length; c, actual screw-head thickness; shortening = b  ×  c/a. FNS, femoral neck system.

Hip function was assessed using the Harris Hip Score (HHS), and pain was assessed using a visual analogue scale (VAS), at 1, 3, 6, and 12 months postoperatively. Complications included surgical-site infection, lower-extremity deep vein thrombosis, implant backing out, implant breakage, and femoral head necrosis.

### Statistical analysis

Statistical analysis was performed in R version 4.2. Continuous variables are expressed as mean and standard deviation, and categorical variables as number (percentage). Normality of continuous variables was assessed before choosing parametric or nonparametric tests. Normality was assessed using the Shapiro–Wilk test and visual inspection of Q-Q plots. A Shapiro–Wilk P value > 0.05 together with an approximately linear Q-Q plot was considered to indicate normality; otherwise, nonparametric tests were used. Before matching, continuous variables were compared using independent-samples t tests and categorical variables using the chi-square test or Fisher exact test, as appropriate. After matching, paired continuous data were compared using paired t tests when distributional assumptions were satisfied; paired binary or multinomial categorical data were compared using the McNemar exact test or Stuart-Maxwell test, as appropriate. Standardized mean differences (SMDs) were used to evaluate covariate balance, with an absolute SMD < 0.10 indicating adequate balance.

Because this was a retrospective study and all eligible patients during the study period were included, no *a priori* sample-size calculation was performed. After propensity score matching, 67 pairs were available for analysis. A *post hoc* paired t-test power assessment based on the observed primary-endpoint difference in 12-month femoral neck shortening (−1.62 mm; 95% CI, −2.06 to −1.18) indicated >99% power at a two-sided alpha level of 0.05; this calculation does not imply adequate power for uncommon complications. For perioperative and radiographic outcomes, normally distributed continuous variables were compared using paired t tests. Guidewire adjustments and length of hospital stay were non-normally distributed and were compared using the Wilcoxon signed-rank test. Fracture-healing time, femoral neck shortening, and neck-shaft angle change were compared using paired t tests; reduction quality was compared using the Stuart-Maxwell test; and 12-month union and moderate-shortening rates were compared using the McNemar test. HHS and VAS values at each follow-up were compared using paired analyses. Overall group, time, and group-by-time effects were analyzed using a linear mixed-effects model that included matched pair as a fixed effect and used patient-level clustering with robust standard errors. Matched complication outcomes were compared using the McNemar or exact test, as appropriate. No formal adjustment for multiple comparisons was applied; secondary and safety analyses were therefore considered exploratory, and individual P values should be interpreted cautiously. A two-sided P < 0.05 was considered statistically significant.

## Results

### Baseline characteristics before matching

All 172 patients had a minimum follow-up of 12 months. The mean follow-up duration was 17.03 ± 2.88 months, with a median of 16.65 months (IQR, 14.88–19.23 months) and a range of 12.0–24.8 months. The mean follow-up durations were 17.10 ± 2.69 months in the FNS plus CCS group and 16.96 ± 3.06 months in the FNS-alone group.Before matching, the Pauwels angle was greater in the FNS plus CCS group than in the FNS-alone group (54.68 ± 6.24° vs. 52.49 ± 7.04°; *P* = 0.032; |SMD| = 0.329). The proportion of Pauwels type III fractures was also higher in the FNS plus CCS group (78.0% vs. 64.4%; *P* = 0.050; |SMD| = 0.304). The injury-to-surgery interval, BMI, and age showed smaller imbalances (|SMD| = 0.232, 0.183, and 0.154, respectively). Other baseline characteristics were similar between groups (Table 1).

**Table 1:** Baseline characteristics of the unmatched cohorts.

Variable	FNS + CCS (*n* = 82)	FNS alone (*n* = 90)	*P value*	|SMD|
Age, years	44.91 ± 9.19	46.32 ± 9.12	0.315	0.154
Male sex	57 (69.5%)	63 (70.0%)	0.945	0.011
BMI, kg/m^2^	23.96 ± 2.81	23.45 ± 2.71	0.233	0.183
Mechanism of injury			0.675	0.120
Traffic accident	37 (45.1%)	46 (51.1%)	-	
Fall from height	32 (39.0%)	33 (36.7%)	-	
Other	13 (15.9%)	11 (12.2%)	-	
Injury-to-surgery interval, h	39.28 ± 17.10	35.32 ± 16.97	0.130	0.232
Left-sided fracture	40 (48.8%)	44 (48.9%)	0.989	0.002
Pauwels angle,°	54.68 ± 6.24	52.49 ± 7.04	0.032	0.329
Pauwels type III	64 (78.0%)	58 (64.4%)	0.050	0.304
Posterior cortical comminution	41 (50.0%)	44 (48.9%)	0.884	0.022

Data are presented as mean ± standard deviation or number (percentage). BMI, body mass index; CCS, cannulated compression screw; FNS, femoral neck system; SMD, standardized mean difference. An absolute SMD < 0.10 indicates adequate covariate balance.

### Baseline characteristics after matching

After matching, all prespecified baseline characteristics were well balanced between groups (Table 2). All absolute SMDs were < 0.10, with a maximum value of 0.068. Changes in propensity score distributions and covariate balance are shown in Figure 4.

**Table 2:** Baseline characteristics of the propensity score-matched cohorts.

Variable	FNS + CCS (*n* = 67)	FNS alone (*n* = 67)	*P value*	|SMD|
Age, years	45.03 ± 9.83	45.22 ± 8.82	0.909	0.021
Male sex	46 (68.7%)	45 (67.2%)	1.000	0.032
BMI, kg/m^2^	23.69 ± 2.80	23.54 ± 2.74	0.748	0.055
Mechanism of injury			1.000	0.000
Traffic accident	32 (47.8%)	32 (47.8%)	-	
Fall from height	25 (37.3%)	25 (37.3%)	-	
Other	10 (14.9%)	10 (14.9%)	-	
Injury-to-surgery interval, h	37.88 ± 17.55	36.69 ± 17.55	0.663	0.068
Left-sided fracture	32 (47.8%)	32 (47.8%)	1.000	0.000
Pauwels angle,°	53.58 ± 5.98	53.86 ± 6.79	0.741	0.044
Pauwels type III	49 (73.1%)	48 (71.6%)	1.000	0.033
Posterior cortical comminution	32 (47.8%)	33 (49.3%)	1.000	0.030

Data are presented as mean ± standard deviation or number (percentage). BMI, body mass index; CCS, cannulated compression screw; FNS, femoral neck system; SMD, standardized mean difference. An absolute SMD < 0.10 indicates adequate covariate balance.

**Figure 4:**
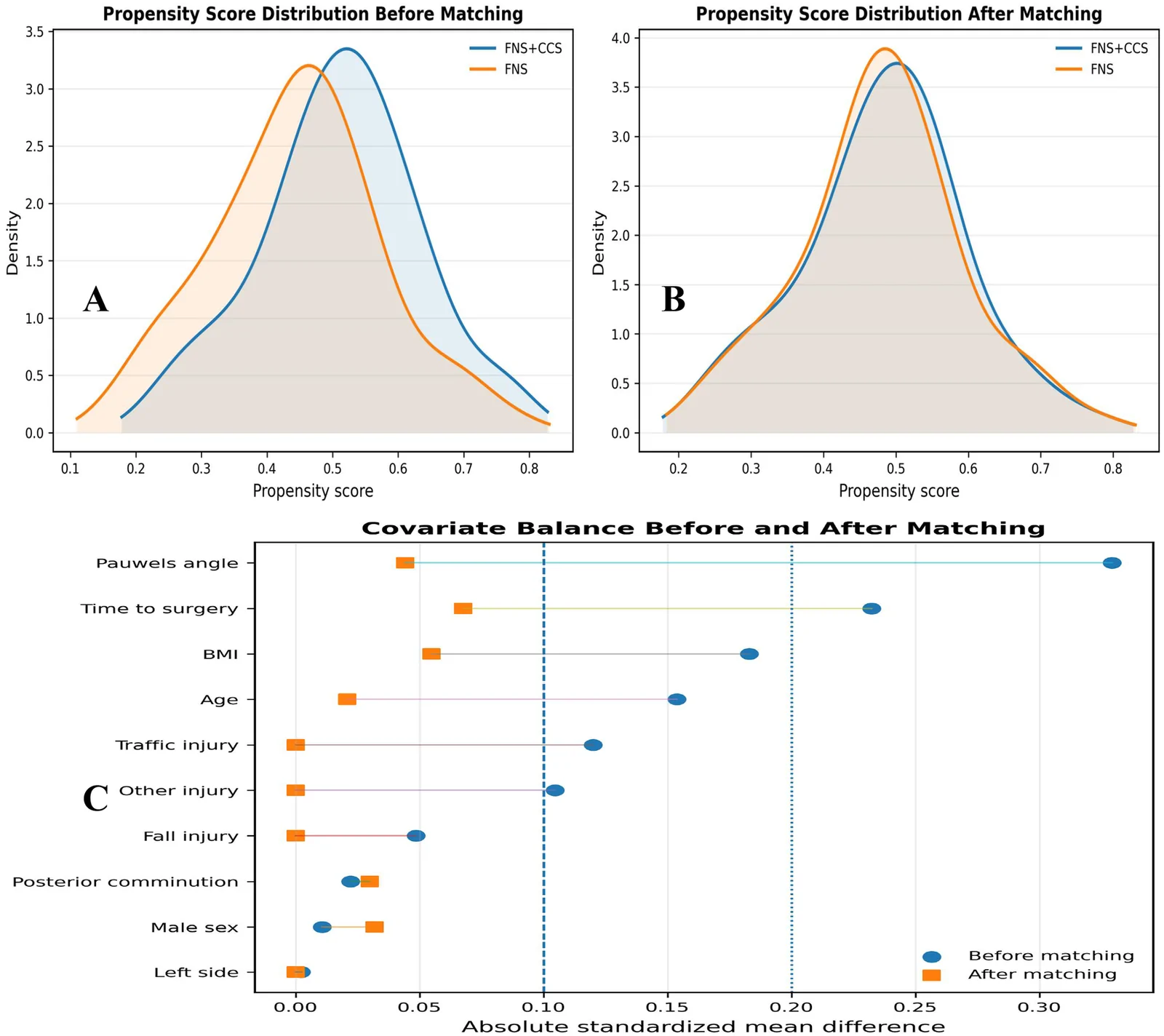
Propensity score distributions and covariate balance. **(A)** Propensity score distributions before matching. **(B)** Propensity score distributions after matching. **(C)** Love plot showing absolute standardized mean differences before and after matching. The dashed line indicates the prespecified balance threshold of 0.10. CCS, cannulated compression screw; FNS, femoral neck system.

### Perioperative and rehabilitation outcomes

The FNS plus CCS group had a longer operative time, more fluoroscopic exposures, more guidewire adjustments, and a longer total incision than the FNS-alone group. The paired mean differences were 13.00 min, 4.22 exposures, 0.67 adjustments, and 0.40 cm, respectively (all P < 0.05). Intraoperative blood loss and length of hospital stay did not differ significantly. Full weight-bearing was achieved earlier in the FNS plus CCS group (10.74 ± 1.42 vs. 12.47 ± 1.34 weeks; paired mean difference, −1.74 weeks; P < 0.001) (Table 3).

**Table 3:** Perioperative and rehabilitation outcomes after propensity score matching.

Outcome	FNS + CCS	FNS alone	Paired mean difference	95% CI	*P value*
Operative time, min	81.67 ± 7.62	68.67 ± 7.51	13.00	10.25 to 15.75	<0.001
Blood loss, mL	79.66 ± 19.76	76.09 ± 19.17	3.57	−2.95 to 10.08	0.278
Fluoroscopic exposures, n	20.25 ± 2.72	16.03 ± 2.72	4.22	3.25 to 5.20	<0.001
Guidewire adjustments, n	1.99 ± 0.79	1.31 ± 0.70	0.67	0.41 to 0.93	<0.001
Total incision length, cm	6.59 ± 0.85	6.19 ± 0.72	0.40	0.14 to 0.66	0.003
Hospital stay, days	6.99 ± 1.21	7.25 ± 1.30	−0.27	−0.71 to 0.18	0.248
Time to full weight-bearing, weeks	10.74 ± 1.42	12.47 ± 1.34	−1.74	−2.23 to −1.24	<0.001

Mean differences were calculated as FNS + CCS minus FNS alone. Normally distributed continuous variables were compared using paired t tests; guidewire adjustments and hospital stay were compared using the Wilcoxon signed-rank test. CI, confidence interval; CCS, cannulated compression screw; FNS, femoral neck system.

### Radiographic outcomes

Fracture-healing time was shorter in the FNS plus CCS group than in the FNS-alone group (4.69 ± 0.52 vs. 5.25 ± 0.43 months; paired mean difference, −0.56 months; P < 0.001). At 12 months, femoral neck shortening was also smaller in the FNS plus CCS group (4.46 ± 1.35 vs. 6.08 ± 1.31 mm; paired mean difference, −1.62 mm; P < 0.001; Figure 5). The incidence of moderate shortening (5-10 mm) was lower with FNS plus CCS (34.3% vs. 73.1%; P < 0.001). Changes in the neck-shaft angle, reduction-quality grade, and 12-month union rate (100.0% vs. 95.5%) did not differ significantly between groups (*P* = 0.470, 0.302, and 0.250, respectively) (Table 4).

**Figure 5:**
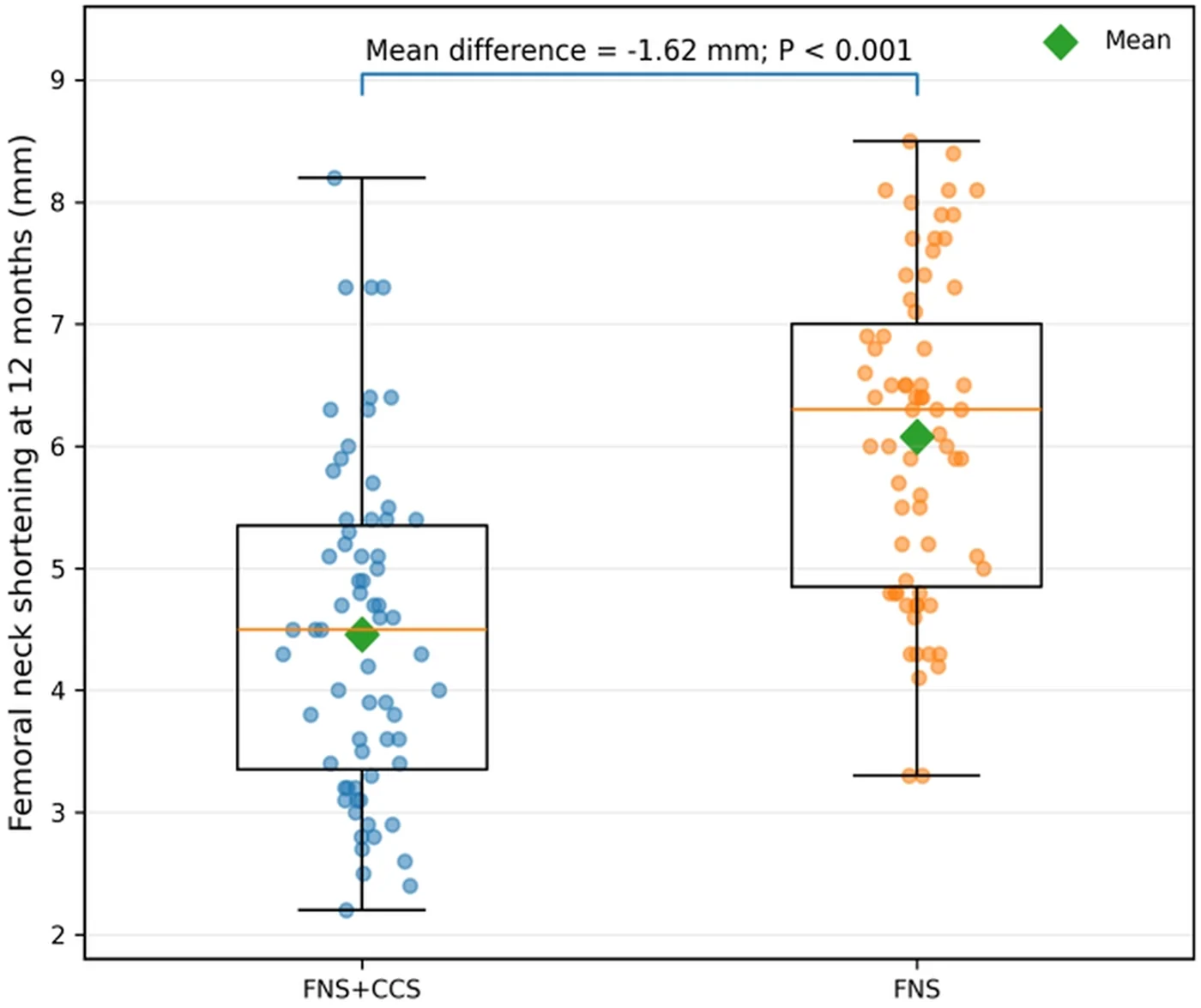
Femoral neck shortening at 12 months. Box-and-whisker plots with individual patient values show less shortening in the FNS plus CCS group than in the FNS-alone group (paired mean difference, −1.62 mm; P < 0.001). Green diamonds indicate group means. CCS, cannulated compression screw; FNS, femoral neck system.

**Table 4:** Radiographic outcomes after propensity score matching.

Outcome	FNS + CCS	FNS alone	Effect estimate	95% CI	*P value*
Fracture-healing time, months	4.69 ± 0.52	5.25 ± 0.43	−0.56	−0.75 to −0.37	<0.001
Femoral neck shortening at 12 months, mm	4.46 ± 1.35	6.08 ± 1.31	−1.62	−2.06 to −1.18	<0.001
Neck-shaft angle change at 12 months,°	2.44 ± 0.92	2.54 ± 0.81	−0.10	−0.36 to 0.17	0.470
Reduction quality, excellent/good/poor	52/13/2	59/6/2	-	-	0.302
Fracture union at 12 months	67 (100.0%)	64 (95.5%)	-	-	0.250
Moderate shortening (5-10 mm)	23 (34.3%)	49 (73.1%)	-	-	<0.001

Effect estimates for continuous outcomes are paired mean differences (FNS + CCS minus FNS alone). Healing time was calculated only among fractures that united; no values were imputed for nonunion. Reduction quality was compared using the Stuart-Maxwell test. CI, confidence interval; CCS, cannulated compression screw; FNS, femoral neck system.

### Functional outcomes

HHS increased over time in both groups. Scores did not differ significantly at 1 or 3 months (*P* = 0.776 and 0.526, respectively), but were higher in the FNS plus CCS group at 6 and 12 months, with paired mean differences of 4.88 and 4.81 points (both P < 0.001). VAS scores did not differ at 1, 3, or 6 months (all P > 0.05), but were lower in the FNS plus CCS group at 12 months (paired mean difference, −0.45 points; P < 0.001). Repeated-measures analyses showed significant group-by-time interactions for both HHS and VAS (both P < 0.001), indicating different trajectories of functional and pain recovery (Table 5 and Figure 6).

**Table 5:** Harris hip score and visual analogue scale outcomes after propensity score matching.

Outcome	Follow-up	FNS + CCS	FNS alone	Paired mean difference	95% CI	*P value*
HHS	1 month	49.16 ± 3.99	48.96 ± 4.04	0.21	−1.25 to 1.67	0.776
	3 months	68.67 ± 4.20	68.58 ± 3.56	0.09	−1.22 to 1.40	0.526
	6 months	83.01 ± 3.31	78.13 ± 3.00	4.88	3.67 to 6.09	<0.001
	12 months	90.31 ± 3.47	85.51 ± 2.90	4.81	3.73 to 5.88	<0.001
VAS	1 month	4.74 ± 0.63	4.87 ± 0.58	−0.12	−0.31 to 0.07	0.208
	3 months	2.79 ± 0.62	2.72 ± 0.59	0.07	−0.17 to 0.30	0.569
	6 months	1.33 ± 0.55	1.36 ± 0.45	−0.03	−0.20 to 0.13	0.693
	12 months	0.57 ± 0.35	1.02 ± 0.35	−0.45	−0.57 to −0.32	<0.001

Time-point comparisons used paired analyses after matching. Overall repeated-measures effects were analyzed using a linear model including matched-pair fixed effects and patient-level clustered robust standard errors. CCS, cannulated compression screw; FNS, femoral neck system; HHS, Harris Hip Score; VAS, visual analogue scale; CI, confidence interval.

**Figure 6:**
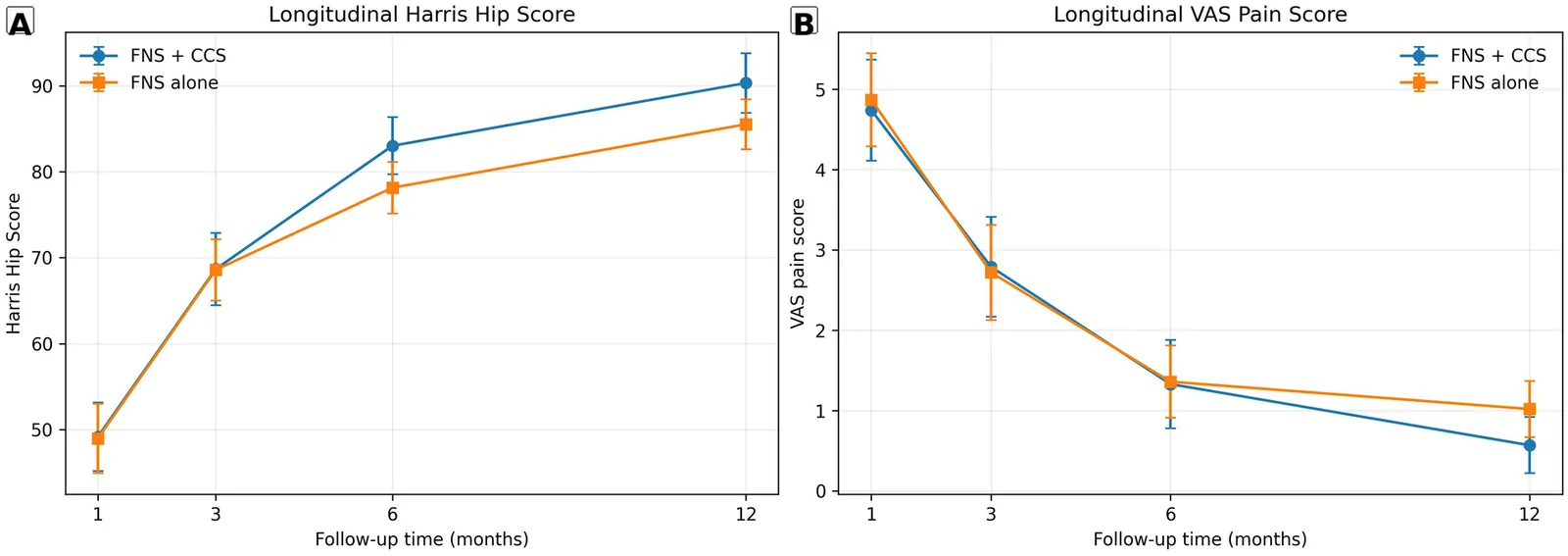
Longitudinal hip function and pain outcomes. **(A)** Harris Hip Score and **(B)** visual analogue scale pain score at 1, 3, 6, and 12 months. Points represent means and error bars represent standard deviations. Significant group-by-time interactions were observed for both outcomes (P < 0.001). CCS, cannulated compression screw; FNS, femoral neck system.

### Postoperative complications

No surgical-site infection, deep vein thrombosis, implant backing out, or implant breakage occurred in the FNS plus CCS group; one patient (1.5%) developed femoral head necrosis. In the FNS-alone group, surgical-site infection occurred in one patient (1.5%), deep vein thrombosis in two (3.0%), implant backing out in two (3.0%), and femoral head necrosis in three (4.5%); no implant breakage occurred. Any complication occurred in 1 of 67 patients (1.5%) in the FNS plus CCS group and 7 of 67 (10.4%) in the FNS-alone group. Among matched pairs, there was 1 discordant pair with a complication only in the FNS plus CCS group and 7 discordant pairs with a complication only in the FNS-alone group, corresponding to a conditional matched odds ratio of 0.14 (exact 95% CI, 0.003-1.11). The difference did not reach statistical significance (continuity-corrected McNemar *χ*^2^ = 3.125; exact *P* = 0.070) (Table 6). The wide confidence interval indicates substantial imprecision; absence of statistical significance should not be interpreted as evidence of equivalence or no safety difference.

**Table 6:** Postoperative complications after propensity score matching.

Complication	FNS + CCS	FNS alone	*χ* ^2^	*P value*
Surgical-site infection	0 (0.0%)	1 (1.5%)		
Deep vein thrombosis	0 (0.0%)	2 (3.0%)		
Implant backing out	0 (0.0%)	2 (3.0%)		
Implant breakage	0 (0.0%)	0 (0.0%)		
Femoral head necrosis	1 (1.5%)	3 (4.5%)		
Any complication	1 (1.5%)	7 (10.4%)	3.125	0.070

CCS, cannulated compression screw; FNS, femoral neck system. The overall complication rate was compared using a continuity-corrected McNemar test; the exact P value is reported. For any complication, the conditional matched odds ratio (FNS plus CCS vs. FNS alone) was 0.14 (exact 95% CI, 0.003-1.11). The wide confidence interval reflects the small number of events and substantial uncertainty.

## Discussion

Garden IV fractures are the most serious type of injuries to the femoral neck in younger and middle aged individuals and are often due to trauma with high energy (14).Apart from the fact that there is complete displacement and significant shear force, disruption of the terminal branches of the medial femoral circumflex artery also pose a high risk of ischemic necrosis. Preserving the hip is thus a priority, but traditional internal fixation may not consistently provide anatomic restoration, mechanical stability, and salvage of the residual blood flow (15).FNS incorporates both axial and torsional stability using a sliding bolt and an anti-rotational screw respectively and was found to be more stable in both axial and torsional directions compared to conventional cannulated screws (16).Nevertheless, the traditional two-component FNS construct may not be adequate on multi-directional rotational instability and shortened Garden IV fractures. Wang et al. (15)reported that after FNS fixation 39.1 percent of patients experienced shortening of the femoral neck, with 17.2 percent being severely shortened. Fan et al. (17) determined that the addition of a cannulated screw to the FNS decreased shortening and enhanced HHS in biomechanical and clinical studies. Freehand fluoroscopy can extend surgery time, raise the amount of radiation received and have the potential to cause injury to the remaining blood supply to the femoral head (18).Robotics navigation involves three dimensional preoperative planning coupled with precise intraoperative implementation and it can attain less than one millimeter accuracy in placing (14, 19, 20).

During this propensity score matched study, there was an increase in operative time, fluoroscopic exposures, guidewire adjustments, and incision length when a CCS was added. These findings indicate that creating the multiplanar fixation configuration is technically more demanding.The combined group achieved full weight-bearing earlier than the FNS-alone group. However, this finding should be interpreted cautiously because progression of weight-bearing was individualized by surgeons who were aware of the fixation construct; therefore, the observed difference may reflect both perceived construct stability and surgeon rehabilitation preference (21). Significantly more important is that the combined group had a shorter healing time, less femoral neck shortening at 12 months, and a significantly smaller rate of moderate-shortening. The results are consistent with the findings of Yu et al. (22) and confirm finite element predictions that an extra screw reduces stress concentration and fragment displacement. Mechanistically, the extra CCS probably increases the fixation area and resistance to rotation, shear, and varus collapse in a plane which is not entirely controlled by the standard FNS.

Functional recovery also differed later in follow-up: HHS scores were higher at 6 and 12 months, and VAS pain scores were lower at 12 months in the combined group. However, the clinical magnitude of these differences should be interpreted cautiously. At 12 months, the between-group differences were 4.81 points for HHS and 0.45 points for VAS. Published MCID estimates for HHS or modified HHS vary substantially across hip-related populations and study methods, but are generally greater than the HHS difference observed in the present study. Similarly, commonly reported MCID values for VAS pain are approximately 1.5–1.8 points on a 0–10 scale, exceeding the observed 0.45-point difference. Therefore, although these differences were statistically significant, they may represent only modest patient-perceived benefits. Moreover, MCID thresholds specifically validated for young and middle-aged patients with Garden IV femoral neck fractures treated with internal fixation are not well established, limiting direct interpretation. These secondary functional and pain outcomes should therefore be regarded as supportive rather than definitive evidence of clinically meaningful benefit.The significant group-by-time interactions suggest that the trajectories diverged later during rehabilitation rather than immediately after surgery. Reduced femoral neck shortening could plausibly help preserve abductor mechanics and hip function (15). However, because no formal multiplicity adjustment was applied, these secondary functional findings should be regarded as exploratory. Any complication occurred in 1.5% of the FNS plus CCS group and 10.4% of the FNS-alone group, but the exact P value was 0.070 and the conditional matched odds ratio was 0.14 with a wide exact 95% CI of 0.003-1.11. Thus, the absence of statistical significance does not demonstrate equivalence or absence of a safety difference, and the study was not adequately powered to evaluate uncommon complications such as femoral head necrosis or implant failure. The 10.4% complication rate in the FNS-alone group was of the same order as the 16.1% early failure rate reported for FNS fixation in a previous study (23). Moreover, because femoral head necrosis may become apparent beyond 12 months, the between-group necrosis rates observed in this study should be regarded as descriptive only and cannot establish a difference in long-term femoral head necrosis risk.

This study has several limitations. First, its retrospective design leaves the possibility of residual and unmeasured confounding despite propensity score matching; prospective studies are needed. Second, although all patients had at least 12 months of follow-up and the mean overall follow-up duration was approximately 17 months, the standardized comparative radiographic and functional outcomes were assessed at 12 months. Moreover, the overall follow-up duration remained relatively short for detecting late femoral head necrosis or establishing long-term implant durability in younger patients.Third, this was a single-center study with a modest matched sample, and all operations were performed by the same experienced team using one robotic navigation platform, which may limit generalizability. Fourth, rehabilitation timing was individualized by surgeons who were aware of the fixation construct, and formal blinding of outcome assessors was not documented; this may have influenced time to full weight-bearing and other clinician-sensitive outcomes. Fifth, interobserver and intraobserver ICCs for femoral neck shortening were not available, and residual error from radiographic rotation or projection may persist despite calibration to known implant dimensions. Finally, no formal correction for multiple comparisons was applied, and the small number of complication events resulted in imprecise safety estimates; therefore, secondary and safety findings should be interpreted cautiously.

## Conclusions

In this propensity score-matched retrospective cohort, robot-assisted FNS fixation combined with an additional CCS in a multiplanar configuration was associated with less femoral neck shortening, shorter fracture-healing time, earlier full weight-bearing, and better later functional recovery than robot-assisted FNS fixation alone, but at the cost of longer operative time and greater fluoroscopic and guidewire-adjustment burden. The complication analysis was underpowered and, with only 12 months of follow-up, cannot establish equivalence, a safety advantage, or any difference in long-term femoral head necrosis risk.These findings are hypothesis-generating and suggest that the combined construct may be a potential hip-preserving option for Garden IV femoral neck fractures in young and middle-aged adults. Prospective multicenter studies with longer follow-up and standardized blinded outcome assessment are warranted.

## Data Availability

The original contributions presented in the study are included in the article/Supplementary Material, further inquiries can be directed to the corresponding author.

## References

[1] Sun K Xu G Liu J Chen L Chen G Wan H. Fns as a more satisfactory surgical option for young and middle-aged patients with femoral neck fractures. Sci Rep. (2025) 15(1):35998. 10.1038/s41598-025-19883-x 41093974 PMC12528660

[2] Zhang C Hong H Ding Z Chen Z Zheng Z Zhang H. Fracture classification and coronal plane position of the bolt may affect prognosis after femoral neck system surgery for femoral neck fractures. BMC Surg. (2024) 24(1):411. 10.1186/s12893-024-02692-w 39710644 PMC11664875

[3] Zhang W Yang N Zhu X Lu X Cheng J. Comparative efficacy of the femoral neck system versus cannulated compression screws in young patients with femoral neck fractures: a systematic review and meta-analysis. Front Surg. (2025) 12:1626320. 10.3389/fsurg.2025.1626320 41141692 PMC12546020

[4] Zhuang K Wu J Yang Y Bai T Li B. Comparison of clinical efficacy between the femoral neck system and cannulated screws in pauwels type III femoral neck fractures: a meta-analysis. J Back Musculoskelet Rehabil. (2025) 38(1):71–82. 10.1177/10538127241296340 39970468

[5] Yusufu A Haibier A Abulaiti A Jian R. Finite element analysis of the femoral neck system combined with a cannulated screw for pauwels type II femoral neck fractures in young and middle-aged adults. Chin J Tissue Eng Res. (2025) 29(15):3095–3100. in Chinese. 10.12307/2025.112.

[6] Silamujiang M Reheman Y Ren Z Yusufu A Jian R Jian W Finite element analysis of three internal fixation methods for pauwels type III femoral neck fractures with anteromedial bone defects. Chin J Tissue Eng Res. (2025) 29(27):5721–5727. in Chinese. 10.12307/2025.195

[7] Zhang Z Qin Q Yusufu A Yuzhe L Reheman Y Jian R. Biomechanical analysis of three internal fixation strategies for pauwels type III femoral neck fractures in young adults. Chin J Tissue Eng Res. (2025) 29(33):7102–7108. in Chinese. 10.12307/2025.849

[8] Yusup T Yusufu A Ran J. Short-term efficacy of femoral neck system fixation combined with cannulated screws at different positions for unstable femoral neck fractures. J Trauma Surg. (2024) 26(7):534–541. in Chinese. 10.3969/j.issn.1009-4237.2024.07.011

[9] Dai Y Wang K Shen G Chen Y Hu A Jin Q. TiRobot-assisted versus freehand femoral neck system placement in the treatment of femoral neck fractures: a systematic review and meta-analysis. J Robot Surg. (2025) 19(1):43. 10.1007/s11701-024-02204-1 39755993

[10] Zheng S Lin D Chen P Lin C Chen B Zheng K. Comparison of femoral neck shortening after femoral neck system and cannulated cancellous screw fixation for displaced femoral neck fractures in young adults. Injury. (2024) 55(6):111564. 10.1016/j.injury.2024.111564 38640596

[11] Sepehri A Martinson J Marchand LS Riedel MD McAlister IP O'Hara NN. Measuring lateral screw protuberance is a clinically accurate method for quantifying femoral neck shortening. J Orthop Trauma. (2020) 34(11):600–605. 10.1097/BOT.0000000000001806 33065661

[12] Xia X Liu Z. Measurement of femoral neck shortening after cannulated screw fixation in older patients and its effect on hip function. Chin J Orthop Trauma. (2014) 16(8):651–655. in Chinese. 10.3760/cma.j.issn.1671-7600.2014.08.002

[13] Li W Yu X Pang Q. Research progress on femoral neck shortening after internal fixation of femoral neck fractures. Chin J Orthop. (2016) 36(6):378–384. in Chinese. 10.3760/cma.j.issn.0253-2352

[14] Wang X Zhang Y Lou L Xu L Fei W Dai J. Robotic-assisted systems for the safe and reliable treatment of femoral neck fractures: a retrospective cohort study. J Orthop Surg Res. (2023) 18(1):633. 10.1186/s13018-023-04070-3 37641097 PMC10463292

[15] Wang K Lin D Chen P Lin C Feng T Liu J. Incidence and factors influencing neck shortening after screw fixation of femoral neck fractures with the femoral neck system. J Orthop Surg Res. (2023) 18(1):317. 10.1186/s13018-023-03787-5 37095563 PMC10127061

[16] Aiheng YL. Comparative efficacy of the femoral neck system and cannulated screws for garden III-IV femoral neck fractures [dissertation] (2025). in Chinese.

[17] Fan J Hao Y Cao Y Cui Z Lv Y Zhou F. Is an additional cannulated screw necessary for unstable femoral neck fractures with comminuted posteromedial cortex treated with femoral neck system fixation? A biomechanical and clinical study. Front Bioeng Biotechnol. (2025) 13:1658728. 10.3389/fbioe.2025.1658728 41323468 PMC12660210

[18] Cong Y Wen P Duan Y Huang H Zhuang Y Wang P. Orthopedic robot-assisted femoral neck system in the treatment of femoral neck fracture. J Vis Exp. (2023):(193). 10.3791/64267 36939232

[19] Tang S Wang B Fan L Li X-b Gao Q Peng Q. Clinical efficacy of robot-navigated femoral neck system fixation for femoral neck fractures. Orthopaedics. (2022) 13(6):511–515. in Chinese. 10.3969/j.issn.1674-8573.2022.06.007

[20] Li Y Wang Y Dong B Yang P Sun Y Zhou L. Comparison of rehabilitation outcomes between robot-assisted and freehand screw placement in the treatment of femoral neck fractures: a systematic review and meta-analysis. BMC Musculoskelet Disord. (2024) 25(1):208. 10.1186/s12891-024-07325-0 38459524 PMC10921808

[21] Liu G Yang C Wang R Tang J Wu H Wei L. Application of a robot navigation system for insertion of the femoral neck system in the treatment of femoral neck fracture. BMC Musculoskelet Disord. (2024) 25(1):47. 10.1186/s12891-024-07172-z 38200451 PMC10777528

[22] Yu X Zhang J Li Y-Z Li X-K Chen F-C Hu H-K. Femoral neck system with an additional cannulated screw for the treatment of elderly femoral neck fractures: a retrospective study. Front Bioeng Biotechnol. (2025) 13:1688008. 10.3389/fbioe.2025.1688008 41480585 PMC12753929

[23] Chen L Jiang JB Ma H Duan X Chen JL. Factors associated with early failure of the femoral neck system in patients with femoral neck fractures. BMC Musculoskelet Disord. (2023) 24(1):912. 10.1186/s12891-023-06994-7 38012667 PMC10680198

